# The Influence Mechanism of Owners’ Safety Management Behavior on Construction Workers’ Safety Citizenship Behavior

**DOI:** 10.3390/bs13090721

**Published:** 2023-08-29

**Authors:** Lei Zhang, Yuanxin Liu, Zhenwei Chu

**Affiliations:** 1 School of Management Engineering, Shandong Jianzhu University, Jinan 250101, China; zhlei06@sdjzu.edu.cn (L.Z.); 2022025224@stu.sdjzu.edu.cn (Y.L.); 2 School of Management, Shanghai University, Shanghai 200444, China

**Keywords:** owners’ safety management, safety citizenship behavior, work engagement, power distance

## Abstract

The safety citizenship behavior (SCB) of construction workers can improve project safety performance. This study explored how construction company owners’ safety management behavior contributes to the development and encouragement of SCB. It combined the Job Demands–Resources (JD-R) and Stimulus–Organism–Response (SOR) theories to propose relevant hypotheses and develop a theoretical model to examine the effect of owners’ safety management behavior on construction workers’ SCB. Data from 534 construction workers were collected through questionnaires. Confirmatory Factor Analysis (CFA) and the Structural Equation Model (SEM) were used for empirical analysis. It was found that the owner’s safety management behavior positively affected the construction workers’ SCB. In particular, work engagement played an intermediary role, while power distance exhibited a moderating effect. A few noteworthy findings are that proactive safety behavior is significantly positively influenced by organization and coordination, prosocial safety behavior is significantly positively influenced by safety funding investment, and high power distance is not always bad for construction workers’ safety citizenship behavior. By determining the connection between owners’ safety management behavior and construction workers’ SCB, this study offered a fresh perspective on promoting construction workers’ proactive behavior and put forward suggestions for owners to improve project safety management.

## 1. Introduction

Construction is considered one of the most dangerous industries, with higher death rates than in any other industry [1]. The construction industry in many countries experiences a significant number of safety incidents, with the US Bureau of Labor Statistics reporting 971 deaths in the construction industry alone in 2017 [2]. In South Korea, the 2020 Industrial Accident Report shows that the construction industry was responsible for 24,617 work-related injuries and 458 fatalities, accounting for 26.6% and 51.9% of total accidents, respectively [3]. In mainland China, statistics from the China Construction Safety Supervision Information System show that there were 689 housing and municipal engineering production safety accidents and 794 deaths in 2020. Unsafe behavior by individuals is the cause of most serious accidents [4]. Because construction workers are the ones who execute engineering operations and contribute to accidents, their behavior toward safety plays a very important role in engineering safety management [5]. Despite numerous studies on the safety behaviors of construction workers over the years, the construction industry is still plagued by accidents and injuries and its safety performance needs to be improved [6,7,8,9]. Some scholars and practitioners have realized that safety citizenship behavior (SCB) plays an important and positive role in organizational safety, especially in construction, because it encourages a safety-oriented behavior and prevents accidents [10,11,12,13,14,15]. SCB refers to voluntary behavior that construction workers spontaneously display to improve the overall safety performance of other workers and organizations. This behavior includes assisting workers in safety improvement, actively reporting safety hazards, actively supervising safety, actively learning about safety, and proposing safety improvement suggestions, which are not mandated by the organization and its leaders. Therefore, engaging in SCB can lead to higher levels of safety performance [16]. Studies show that construction company owners’ active involvement in safety management significantly impacts project safety performance. Although owners need not always engage in engineering safety, their attitude towards safety and involvement in safety management play a positive role in improving the safety-oriented behavior of construction workers [17]. Therefore, owners may have an important role in guiding and stimulating the SCB of construction workers.

One study found that individual characteristics and psychological and organizational factors have an impact on the SCB of construction workers [18]. Previous studies on SCB mostly considered employee personality and psychological factors [19,20]. One study conducted research from an organizational perspective, including the organization’s safety atmosphere and level of support [21], while other studies considered the impact of leadership [22,23]. In respect to the research undertaken in other studies, this study considered whether the owner, as the person primarily responsible for safety, can provide individual, psychological, and organizational support for construction workers and create a positive impact on their SCB. However, when it comes to safety management, most owners rely on safety norms to restrict workers’ behavior rather than actively guide workers’ safety-related behavior [18]. As a result, there is an urgent need to transform the attitude of passively restricting workers to actively guiding them to promote the development and encouragement of SCB.

Despite this issue, some researchers have discussed the relationship between owners’ safety management and construction workers’ behaviors. For example, the authors of [24] discussed how owners’ safety management behaviors affect construction workers’ unsafe behaviors. Other studies analyzed the importance and role of owners’ safety management in improving safety performance [17,25,26]. However, current research has paid little attention to the proactive and voluntary behavior of construction workers in terms of safety in the workplace. Similarly, research on the effect of owners’ safety management behavior on SCB is almost non-existent. It remains unclear whether one impacts the other and what mechanism produces this impact. Existing studies have confirmed that some factors, such as work engagement and power distance, play a mediating or moderating role in the owner’s influence on construction workers’ behaviors [27,28]. In construction safety management, workers’ involvement at work and the power distance between them and the owner are considered to be crucial factors affecting safety performance [29,30]. Nevertheless, it remains unclear whether these factors play a mediating or moderating role in the relationship between owners’ safety management behaviors and construction workers’ SCB. Therefore, empirical research is needed to clarify the connection between owners’ safety management behavior, work engagement, power distance, and workers’ SCB.

This study aims to fill the existing research gap by exploring the influence of owners’ safety management behavior on construction workers’ SCB, exploring the multi-dimensional impact relationship between variables, and uncovering the mechanism behind the influence. Furthermore, it aims to explore whether there is a mediating role for work engagement and a moderating role for power distance. This study offers owners a way to help construction workers by providing them with individual and organizational support and guiding their SCB. This, in turn, helps improve the safety performance of the construction industry.

The rest of the study is structured in the following way: Section 2 reviews the relevant literature on owners’ safety management and SCB and identifies the current research gaps and deficiencies. Section 3 introduces the research theory and puts forward research hypotheses. Section 4 introduces the research methods, including questionnaire design and data collection. Section 5 provides empirical analysis by evaluating the reliability and validity of the scale and the established structural equation model and testing the hypotheses. Section 6 discusses and explains the research results and offers suggestions. Finally, Section 7 summarizes the study.

## 2. Literature Review and Research Gaps

### 2.1. Owners’ Safety Management Behavior

Safety management behavior represents a series of working methods and implementation processes to prevent accidents and control accident-related losses [26]. The results of one study [17] show that safety management behaviors such as safety requirements and participation of owners in safety measures have a positive impact on safety performance during construction work. The authors of [31] believed that owner safety management includes eight aspects, namely objectives and assessment, safety organization, inspection and rectification, education and training, safety information, stakeholder management, accident management, and emergency management. To uncover the specific mechanism of the influence of owners’ safety management behaviors on construction workers’ SCB, this study selected four key aspects of owners’ safety management behaviors after reviewing construction industry standards and relevant literature: safety objectives (SO), organization and coordination (OC), safety funding investment (SFI), and safety supervision (SS).

Firstly, owners need to set SO and ensure that all personnel understand the safety objectives and tasks associated with a project. Furthermore, owners should carry out frequent follow-ups to ensure adherence to the objectives. Setting safety objectives and requiring subordinates to firmly adhere to these goals is an important indicator of safety control and performance management [32]. Owners’ expectations of safety objectives will create a favorable corporate safety climate and influence workers’ behavior [33,34]. Secondly, owners should implement OC by organizing and coordinating project safety regulations, safety responsibility systems, and emergency reward mechanisms to improve safety performance and reach safety objectives [35]. Through coordination, the owner also deals with the complex relationships and contradictions among the many parties involved in the construction project. Safety work is no exception, and the owner is required to coordinate the construction project management team and define the safety responsibilities of each party [36]. The third aspect is SFI, which includes equipment maintenance, safety education and training for construction workers, incentives for workers who report risks, etc. The success or failure of the owner’s investment in a project and their reputation heavily rely on construction safety. Therefore, owners should commit to allocating financial resources for construction work and project safety [17]. Finally, SS includes regular investigation and rectification of safety hazards, strict supervision of high-risk construction activities, and punishment for those responsible for accidents. The authors of [37] proposed that owners should participate in safety meetings, site safety supervision, accident investigations, and other safety-related work through their project representatives.

Previous studies related to owners’ safety management in other fields devoted attention to the owners’ attitudes toward safety management. For example, the authors of [38] assessed owners’ behaviors and attitudes toward implementing food safety and quality management systems. The authors of [39] explored the personal views and experiences of cleaning service owners regarding their responsibilities toward managing occupational safety and employee health. Research on owners’ safety management in construction primarily focuses on how to improve the level of owners’ safety management. For instance, the authors of [26] developed a safety management index system suitable for real-estate owners, analyzed the relationship between the safety management system, safety management behavior, and safety management state, and discovered the main factors influencing the relationship. The authors of [40] discussed the influence mechanism of the leadership cultural behavior (LCB) of owners on owners’ safety management and drew up an optimal intervention plan for improving owners’ safety management in small and medium-sized enterprises. Furthermore, some researchers have studied the impact of owners’ safety management behaviors on workers’ behaviors. For example, the authors of [24] examined the influence of owners’ safety management behaviors on the unsafe behaviors of construction workers, considering five aspects. Despite these important pieces of research, scholars have to date paid little attention to the safety management behavior of owners and the active safety behavior of construction workers. Therefore, this area requires thorough research.

### 2.2. Safety Citizenship Behavior of Construction Workers

The concept of SCB was first proposed by the authors of [16], who defined it as an individual’s initiative to ensure that other organization members perform their tasks safely or that they themselves improve the safety of the entire project. Ref. [41] further elaborated on the concept of SCB and described it as a behavior that helps other members and organizations achieve safety objectives and improve safety within the workplace. The research on SCB is based on organizational citizenship behavior (OCB), which refers to the voluntary behavior of employees to perform other tasks, which positively affects the organization [41]. The authors of [16] applied the research on OCB to the security sector and proposed an SCB structure containing six behavior types. Later, scholars improved the structure and included proactive and prosocial safety behaviors. On the one hand, proactive safety behavior (ASB) includes making safety proposals and actively and spontaneously changing safety working methods. On the other hand, prosocial safety behavior (SSB) includes tasks such as assisting fellow workers in performing tasks more safely, showing concern for their welfare, voluntarily participating in organizing safety-related activities, etc. [42,43]. Therefore, scholars created a two-dimensional SCB structure [44,45,46]. The present study also considered the aforementioned SCB sub-dimensions.

Previous studies on SCB have found that the two dimensions of SCB can be encouraged through different means of support [45,46]. Other areas of research on SCB include the influence of leaders and organizations. For instance, the authors of [47] studied the relationship between safety-specific transformational leadership and employee–subordinate SCB. The authors of [48] offered insight into how organizational support affects employees’ SCB. The research on construction workers’ SCB includes individual, psychological, and organizational influences. The authors of [15] studied the drivers of construction workers’ SCB from the perspective of social security capital. Moreover, the authors of [11] explored the impact of three safety stressors (role ambiguity, role conflict, and interpersonal conflict) and safety-specific trust on construction workers’ SCB. However, there is a research gap when it comes to exploring the perspective of owners.

To summarize, research on owners’ safety management behavior and SCB has received attention in a variety of disciplines, with several research findings. However, to date, researchers have given little attention to owners’ safety management behavior and construction workers’ initiative behavior, and there is a paucity of studies on fostering and cultivating construction employees’ safety citizenship behavior from the perspective of owners. Considering the importance of owners in construction projects, it is necessary to explore the influence of owners’ safety management behaviors on the two dimensions of SCB, considering SO, OC, SFI, and SS.

## 3. Theory and Hypotheses

### 3.1. Influence of Owners’ Safety Management Behavior on Construction Workers’ SCB

The theory of Job Demands–Resources (JD-R) has been applied in the research of safety behavior [49]. Safety objectives represent mandatory work requirements for workers. According to the theory of JD-R, when employees are faced with a highly challenging work environment, they tend to set higher expectations for their job, enabling them to effectively use higher job resources and deliver high work performance [50,51]. As a result, the owner’s safety objective management has a positive impact on active SCB. The JD-R model divides any job characteristic into job demand and job resources [52]. Job demand refers to the factors that require an individual to invest energy or money to meet the job requirements, including requirements related to an individual’s physical, mental, and social ability [53]. In their work, the authors of [54] pointed out that in a work environment that focuses on the safety of the organization, employees are more compliant with safety procedures. Therefore, the establishment of project safety objectives is also conducive to the encouragement of prosocial safety behaviors in workers, such as following safety procedures. The unsafe behavior of some workers may not cause harm to others but can have an impact on the safety and efficiency of the entire project. The shared interests of workers who follow shared safety objectives may drive construction workers to help one another and establish prosocial safety behavior. As a result, owners’ safety objective management has a positive effect on both proactive and prosocial safety behaviors of construction workers.

According to the JD-R theory, role conflict, role ambiguity, work overload, and job insecurity negatively affect an individual’s work enthusiasm and energy [55]. In case the owner fails to organize and coordinate the work properly, and if there is a lack of clear division of labor within the team, workers may receive conflicting instructions from multiple superiors, making it difficult to comply with all the instructions simultaneously. Such different expectations can lead to role ambiguity and conflict [56]. In particular, role ambiguity means that individuals have unclear ideas about their role or have difficulty fulfilling their roles in light of given information and resources [57,58]. Conversely, role conflict refers to the disparity between an individual’s perceived role and actual expectations placed upon them, which results from others lacking a common understanding of a specific role [59,60]. When workers are confused about the safety procedures and it becomes difficult for them to distinguish between incorrect and correct procedures, it damages their confidence and dents their enthusiasm in actively helping others achieve safety performance, which is not conducive to prosocial safety behavior [61,62,63]. In situations where role conflicts occur, workers are often faced with dilemmas, making it difficult to make optimal decisions to improve work safety, which affects their proactive safety behavior [64]. In conclusion, negative factors such as role ambiguity and role conflict do not contribute to construction workers’ SCB [11]. What can help reduce the effect of the two factors is the owner organizing and coordinating safety procedures, clarifying the safety responsibilities of all parties, and establishing an effective communication and coordination mechanism. This can also have a positive impact on the proactive and prosocial safety behavior of construction workers.

Job resources include physical, psychological, social, or organizational resources, that have a positive impact on individuals, enabling them to achieve work goals, stimulate individual learning, growth, and development, and reduce work demands, psychological burden, and physical costs [65]. Owners’ investment in safety not only improves the safety of the working environment but also increases job resources, resulting in improved work engagement and positive outcomes [66]. Work autonomy is one of the most important prerequisites for work initiative [67]. There is evidence that workers relate their jobs to positive work experiences, such as performing important high-level tasks and engaging in civic behavior [54,68]. Therefore, safety funding investment is conducive to stimulating construction workers’ proactive safety behavior. In addition, strengthening safety education and training can also help improve the safety awareness of construction workers, cultivate workers’ safety values, enhance workers’ safety capabilities, which are the prerequisites for SCB, and create favorable conditions for them to encourage prosocial behavior in other workers [18]. Therefore, owners’ funding investment in safety has a positive impact on construction workers’ proactive and prosocial safety behavior.

According to Stimulus–Organism–Response (SOR) theory, environmental stimuli have an impact on employees’ emotional responses and subsequent behavior. As the building block of modern cognitive psychology, SOR theory reveals how environmental factors affect an individual’s emotional response, which produces a certain behavior [69]. Stimulus (S) is a driving force in the work environment, having an impact on an employee’s individual cognitive awareness or emotional perception [70]. Organism (O) mainly refers to human emotion and cognition, which represents an internal process and relationship between the stimulus and the final response, i.e., behavior [71]. Response (R) is regarded as the response or behavior following a reaction. It includes psychological reactions (such as attitude) and behavioral responses [69]. Safety performance gains greater importance in a positive safety environment than in a negative one. Moreover, individuals are more likely to broaden their understanding of what it is to be a safety-oriented citizen in a positive safety environment [54]. Safety monitoring can increase workers’ SCB because it influences construction workers’ perceptions of the safety policies, regulations, and procedures of an organization [72]. For example, regular safety inspections and safety hazard investigations encourage construction workers to report safety hazards, discourage unsafe behavior by other workers, and promote prosocial safety behavior. The author of [73] believes that a sense of safety within a team influences construction workers’ motivation and shapes their expectations and perceptions concerning safe or unsafe behavior. In addition, it influences owners to clearly communicate the expected roles of construction workers, fostering their proactive safety behavior. In conclusion, safety supervision has a positive impact on the proactive and prosocial safety behavior of construction workers.

In light of the aforementioned theory, this study proposed the following hypotheses:

**Hypothesis 1** **(H1).** *SO positively influences ASB.*

**Hypothesis 2** **(H2).** *SO positively influences SSB.*

**Hypothesis 3** **(H3).** *OC positively influences ASB.*

**Hypothesis 4** **(H4).** *OC positively influences SSB*.

**Hypothesis 5** **(H5).** *SFI positively influences ASB*.

**Hypothesis 6** **(H6).** *SFI positively influences SSB*.

**Hypothesis 7** **(H7).** *SS positively influences ASB*.

**Hypothesis 8** **(H8).** *SS positively influences SSB*.

### 3.2. Work Engagement

Work engagement refers to an individual’s enthusiastic and wholehearted involvement in their work, which represents a psychological recognition and acknowledgment of their work. The author of [74] proposed that work engagement represents an employee’s ability to exercise control over themselves in an organization and seamlessly integrate themselves into their work roles. They divided work engagement into three dimensions: physical, cognitive, and emotional. Firstly, physical involvement refers to maintaining a high degree of physical involvement when an individual is engaged in work. Cognitive engagement means that individuals have a clear and active perception of their role. They can clearly define their position and responsibilities in the workplace. Finally, emotional engagement reflects an individual’s sense of belonging and identity, which means that an individual keeps in touch with colleagues, superiors, and other people, and feels compassion for them [74]. The three dimensions are relatively independent. However, the higher an individual’s engagement in one dimension, the higher their overall work engagement [66]. Therefore, this study viewed work engagement as a single dimension. The authors of [75] extended this model by incorporating work engagement into the JD-R model. By ensuring sufficient job resources and improving work engagement, a desired positive outcome can be achieved.

The owner’s establishment of a clear and unified safety goal can improve construction workers’ cognition and promote value consistency, which is conducive to improving their work engagement [76]. The owner fosters a cooperative organization in which they coordinate participating parties and effectively communicate the responsibilities of the parties. This positive factor contributes to open communication among construction workers, clarifying their roles and responsibilities, and thereby enhancing work engagement [77]. According to the JD-R theory, safety funding investment can enhance the number of job resources, stimulate employees’ motivation, and improve their work engagement [66]. The owner’s active safety supervision and enforcement of specific safety norms, rules, and regulations can ensure that construction workers perform their production work safely. This can, in turn, improve the physical engagement of workers. Therefore, safety objectives, organization and coordination, safety funding investment, and safety supervision can improve the physical, emotional, and cognitive work engagement of construction workers. It is speculated that they have a positive impact on work engagement.

Work engagement has been recognized as a major driver of positive job performance beyond individual expectations [78]. When construction workers maintain strong physical work engagement, they can put energy into their work, work wholeheartedly, and strive to excel [74]. Therefore, work engagement can stimulate the proactive safety behavior of construction workers. The authors of [79] believed that a high level of work engagement enables individuals to fully invest their energy into their roles and behaviors, and effectively demonstrate their roles. This highly active and awakened state helps construction workers consciously nurture SCB and fulfill work responsibilities. Additionally, it encourages them to participate in safety-related meetings, actively seek safer working methods, and display proactive and prosocial safety behaviors. Moreover, work engagement can enable individuals to connect with others [74]. If construction workers maintain strong emotional work engagement, they can maintain deeper connections with colleagues and superiors. This is conducive to construction workers giving advice to co-workers in terms of safety issues and encouraging them to abide by safety procedures and display prosocial safety behaviors. In conclusion, work engagement can have a positive effect on both proactive and prosocial safety behavior.

Therefore, this study developed the following hypotheses:

**Hypothesis 9 (H9).**  *SO positively influences WE.*

**Hypothesis 10 (H10).**  *OC positively influences WE.*

**Hypothesis 11 (H11).**  *SFI positively influences WE.*

**Hypothesis 12 (H12).**  *SS positively influences WE.*

**Hypothesis 13 (H13).**  *WE positively influences ASB.*

**Hypothesis 14 (H14).**  *WE positively influences SSB.*

**Hypothesis 15 (H15).**  *WE mediates between owner’s safety management behavior and construction worker SCB.*

### 3.3. Power Distance

Power distance (PD) refers to the degree of acceptance and expectation among low-power members regarding the unequal distribution of power in social groups and organizations [80]. It can be divided into four levels: country, organization, team, and individual. In addition, power distance can be divided into leadership power distance and employee power distance according to individual orientation [81]. This study adopted power distance at the individual level, that is, the degree of construction workers’ acceptance of the owner’s unique power attributes. This acceptance forms the power distance perception of construction workers.

The authors of [82] found that high power distance hinders workplace communication. The authors of [83] showed that a superior’s inclination towards power distance positively affects employees’ silent behavior by influencing their sense of usefulness and psychological security. In a high power distance environment, employees usually respect the authority of their superiors. When there is a difference between the superior’s orders and their own opinions, employees usually choose to obey the orders of the superior. Leadership behaviors tend to be centralized and authoritarian, which makes it difficult for employees to act independently [84,85]. Therefore, high power distance does not contribute to the development and encouragement of construction workers’ proactive safety behavior. In contrast, low power distance advocates empowerment, fairness, and democracy. Employees in a low power distance environment exhibit proactiveness, which enhances trust in co-workers and superiors. In addition, low power distance creates a positive atmosphere where employees can make safety suggestions and facilitates freedom of speech [86,87]. It can promote unity within an organization, which encourages construction workers to assist their colleagues in working more safely. Employees in a low power distance environment are willing to establish a relationship based on equality with superiors. The more support from the organization or superior, the better the employees perform their tasks [85].

In light of the aforementioned theory, this study puts forward the following hypothesis:

**Hypothesis 16 (H16).**  *PD moderates the impact of owners’ safety management behavior on construction workers’ SCB.*

**Hypothesis 16a (H16a).**  *PD moderates the effect of SO on the ASB.*

**Hypothesis 16b (H16b).**  *PD moderates the effect of SO on the SSB.*

**Hypothesis 16c (H16c).**  *PD moderates the effect of OC on the ASB.*

**Hypothesis 16d (H16d).**  *PD moderates the effect of OC on the SSB.*

**Hypothesis 16e (H16e).**  *PD moderates the effect of SFI on the ASB.*

**Hypothesis 16f (H16f).**  *PD moderates the effect of SFI on the SSB.*

**Hypothesis 16g (H16g).**  *PD moderates the effect of SS on the ASB.*

**Hypothesis 16h (H16h).**  *PD moderates the effect of SS on the SSB.*

**Figure 1:**
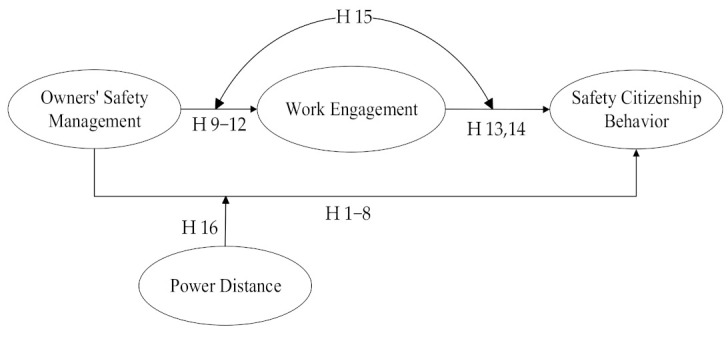
Hypotheses Model.

Based on JD-R theory and SOR theory, this study developed a model of the impact of owners’ safety management behaviors on construction workers’ SCB. The model is shown in Figure 1.

## 4. Methodology

### 4.1. Procedures and Participants

**Figure 2:**
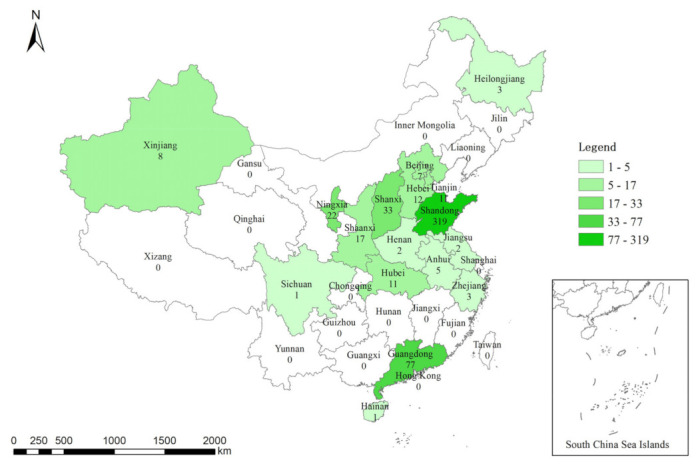
Areas from where the data came shaded according to number of survey responses.

The proposed hypotheses were tested using a comprehensive question-and-answer survey. Firstly, a preliminary questionnaire was developed and sent to 10 construction workers at a construction site in Shandong Province, China, to ensure the readability and accuracy of the questions. Based on the results, the language of the question was simplified so that construction workers could understand the questions and answer them accordingly. Likewise, some questions were deleted to form a formal questionnaire. The formal questionnaire was divided into five parts: (a) Owner’s safety management behavior; (b) Construction workers’ SCB; (c) Work engagement; (d) Power distance; and (e) Demographic characteristics, including respondent age, gender, education level, years of service in the construction industry, and position. Because information on all construction workers cannot be gathered, determining the total number of subjects is problematic, and the survey adopts a non-probabilistic sampling method [88]. The non-probability sampling method has been widely used in the research of construction engineering management [89,90]. Convenience sampling is used in this study because it can obtain a wider survey population and has the advantages of time and cost-effectiveness [91]. This study used the back-translation approach developed by the authors of [92] to translate the scales published in English into Chinese and compare them. This questionnaire survey was conducted online from February to April 2023. The network collected samples from across the country. Questionnaire data were collected by Questionnaire Star, a professional electronic questionnaire survey platform, and construction workers in various locations were contacted and sent questionnaires to invite them to participate in the survey. The majority of the data came from Shandong, Guangzhou, Shanxi, and Ningxia. The data area map is shown in Figure 2.

Questions on owners’ safety management behavior were adapted from the questionnaire developed by the authors of [31]. The data on owners’ safety management behaviors was collected based on the four key aspects mentioned in Section 2.1. This section included 16 statements, e.g., “The Group has detailed and regular security tasks and objectives”. The section about SCB included 12 statements from a study [16] on helping, whistleblowing, voice, and initiating safety-related change, e.g., “I will give advice on work activities related to safety”. The higher the score in this section, the more SCB the respondents had. The section about work engagement adopted a portion of the work input scale developed by the authors of [78], which mainly considers that work engagement is divided into three dimensions: physical, emotional, and cognitive engagement. Statements in this section include “I exert my full effort to my job”. Of course, the higher the score in this section, the higher the work engagement. In this study, power distance represents a part of the scale developed by the authors of [93] and includes four statements, such as “Managers do not need to consult subordinates when making most of the decisions”. All four scales were measured using a 5-point Likert scale and the scores ranged from 1 = “completely disagree” to 5 = “completely agree”. The complete items of the scale are given in Table 1.

To reduce potential bias, participants were informed prior to answering the questionnaire that all information collected would be anonymous and treated confidentially. They were assured that the researchers collecting the data were independent and had no connection to their employers or superiors. Additionally, participants were assured that their responses would be analyzed without revealing their identity and used for academic purposes only. Finally, this study obtained the informed and written consent of all participants.

### 4.2. Data Analysis

After screening the samples, questionnaires with too many missing values, extremely short response times, and zero variance were excluded from the initial 610 responses. As a result, 534 valid responses were taken as samples, resulting in a questionnaire efficiency of 87.5%. The common criterion is that the sample size should be over 10 times the measurement item and it should have no upper limit [94,95,96]. The measurement item in this study was 41, so the sample size can be considered reasonable.

The partial least squares structural equation model (PLS-SEM) is adopted in this study, which is appropriate for theoretical investigation and causality verification [97]. It has the advantage of not requiring a large number of data samples and is appropriate for assessing complex structural equation models [98,99]. SPSS 27.0 and SmartPLS 4.0 software were used to analyze the data in three stages. Firstly, the study carried out a descriptive analysis of samples and variables. Then, the reliability of the scale and the validity of the model were tested. The latter was tested through confirmatory factor analysis (CFA), including convergence and discriminant validity. Finally, hypotheses were tested by determining the relationship between different structures.

## 5. Results

### 5.1. Descriptive Analysis of Samples and Variables

#### 5.1.1. Descriptive Analysis of Samples

Table 2 shows the final demographic results, including respondents’ gender, age, education, years of service, and position. Among the respondents who provided valid data, 54.4% were civil workers (masons, steel workers, paint workers, etc.), 24.5% were decoration workers (plumbers, ventilation technicians, riggers, etc.), and 6.1% were urban construction workers (road workers, sewer workers, landscapers, etc.).

#### 5.1.2. Descriptive Analysis of Variables and Normality Tests Performed on Variables

The mean score of each variable was between 3 and 4. The scale adopted in this study had a positive score of 1–5. Therefore, the cognitive and behavioral levels of the participants in this study were above the medium level in respect to the owners’ safety management behavior, SCB, work engagement, and power distance. Skewness and kurtosis were used to test the normality of each measurement item. According to the standard, the absolute values of skewness coefficients in this study were within 3, while those of kurtosis coefficients were within 10. Therefore, the data had an approximately normal distribution [100].

### 5.2. Reliability and Validity of the Scale

#### 5.2.1. Reliability Analysis

The reliability of each variable was assessed using Cronbach α (CA), which collected the data. Table 3 shows the Cronbach α between the variables. The value of Cronbach α ranged from 0 to 1. The higher the test result value, the higher the reliability. Generally, a value below 0.6 is considered to be unreliable. In such a case, the questionnaire should be redesigned, or the data should be collected again and analyzed. A value higher than 0.7 indicates that the variable shows increased reliability. As the results in Table 3 show, based on the CA score the four aspects of owners’ safety management behavior, construction workers’ SCB, work engagement, and power distance had high reliability [101]. In addition, Cronbach α for each sub-dimension of a variable was appropriately applied. As a result, the scales exhibited good internal consistency and the reliability of variables is guaranteed.

#### 5.2.2. Confirmatory Factor Analysis

The convergence validity (AVE) and composite reliability (CR) of each dimension of the scale variables were further tested in this study. The results are shown in Table 3. Based on data analysis, it can be seen that all dimensions of the scale variable had good convergence validity and combination reliability since all AVE and CR values were greater than 0.5 and 0.7, respectively [102,103].

This study’s discriminant validity meets the Fornell–Larcker criteria. However, the authors of [104] proposed that the robustness of this criterion in evaluating the discriminant validity is still controversial, so this study uses their proposed HTMT ratio as a more adequate alternative to evaluate the discriminant validity of external models. The authors of [104] proposed that if the HTMT value is higher than 0.90, the differential validity is poor, and the more conservative HTMT threshold is 0.85. The results are shown in Table 4. Owners’ safety management behavior and construction employees’ safety citizenship behavior are both related to safety behavior in this study, and the dimensionality concept is somewhat similar. Therefore, as all HTMT values between sub-dimensions are less than 0.9, it can be considered that the scale still has good discriminative effectiveness [103].

### 5.3. Hypothesis Testing and Results

**Figure 3:**
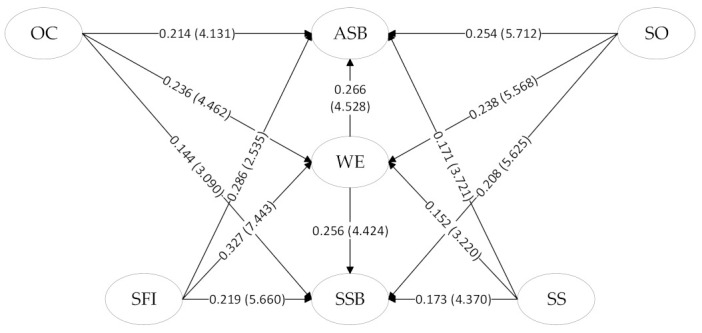
Structural equation model.

SmartPLS 4.0 software was used to analyze the structural equation model based on the aforementioned hypotheses. The software was also used to test the influence of the key aspects of owners’ safety management behavior on different dimensions of construction workers’ SCB and examine the mediating effect of work engagement and the possible moderating effect of power distance on the aspects of ASB and SSB. The test results are shown in Table 5 and the structural relationship is shown in Figure 3.

#### 5.3.1. Model Fitting

According to the results of model fitting, the standardized root mean square residual (SRMR) = 0.036 and is, therefore, less than 0.08, the NFI = 0.876, and the $R^{2}$ values of ASB and SSB were 0.867 and 0.919, respectively. The $Q^{2}$ values of ASB and SSB were 0.656 and 0.691, respectively. In general, an $R^{2}$ of over 0.67 indicates strong explanatory power [95] and if the $Q^{2}$ value is greater than 0.5, the prediction correlation is high [105]. The GoF is 0.776, meaning that the model is able to account for 77.6% of achievable fits [106]. Therefore, it was found that the SEM model had a good fit to the data.

#### 5.3.2. The Influence of Owners’ Safety Management Behaviors on Construction Workers’ SCB

Because all *p*-values were less than 0.05 in the hypotheses test, hypotheses H1–H16 were valid. The four dimensions of owners’ safety management behavior (SO, OC, SFI, and SS) had significant positive effects on the two dimensions of construction workers’ SCB (ASB and SSB), in which work engagement played an intermediary role while power distance moderated the impact of owners’ safety management behavior on construction workers’ SCB. Next, we analyzed the data contained in Table 5. Hypothesis 1 is that SO has a positive effect on ASB. This hypothesis is verified, and the data show that β = 0.254, T = 5.712, *p* < 0.001. Hypothesis 2 is that SO has a positive effect on SSB, and this hypothesis is also verified, as shown by β = 0.208, T = 5.625, *p* < 0.001. Hypothesis 3 is that OC has a positive impact on ASB, and the data show that β = 0.214, T = 4.131, *p* < 0.001, which supports this hypothesis. Hypothesis 4 is supported by the data (β = 0.144, T = 3.09, *p* < 0.01), and OC has a significant positive effect on SSB. Hypothesis 5 is supported by the data (β = 0.286, T = 2.535, *p* < 0.05), and SFI has a significant positive effect on ASB. Hypothesis 6 is supported by the data (β = 0.219, T = 5.66, *p* < 0.001), and SFI has a significant positive effect on SSB. Hypothesis 7 is supported by the data (β = 0.171, T = 3.721, *p* < 0.001), and SS has a significant positive effect on ASB. Hypothesis 8 is supported by the data (β = 0.173, T = 4.37, *p* < 0.001), and SS has a significant positive effect on SSB. Hypothesis 9 is supported by the data (β = 0.238, T = 5.568, *p* < 0.001), and SO has a significant positive effect on WE. Hypothesis 10 is supported by the data (β = 0.236, T = 4.462, *p* < 0.001), and OC has a significant positive effect on WE. Hypothesis 11 is supported by the data (β = 0.327, T = 7.443, *p* < 0.001), and SFI has a significant positive effect on WE. Hypothesis 12 is supported by the data (β = 0.152, T = 3.22, *p* < 0.01), and SS has a significant positive effect on WE. Hypothesis 13 is supported by the data (β = 0.266, T = 4.528, *p* < 0.001), and WE has a significant positive effect on ASB. Hypothesis 14 is supported by the data (β = 0.256, T = 4.424, *p* < 0.001), and WE has a significant positive effect on SSB. Hypothesis 15 is supported by the data (β = 0.221, T = 4.247, *p* < 0.001), and WE had a significant mediating effect between owner safety management behavior (SMB) and SCB. Hypothesis 16 is supported by the data (β = −0.059, T = 3.891, *p* < 0.001), and PD had a significant regulatory effect between SMB and SCB.

Figure 3 shows the structural equation model established according to the hypothesis. In the figure, SO, OC, SFI, and SS are the four sub-dimensions of owner safety management of independent variables, ASB and SSB are the two sub-dimensions of dependent variables SCB, WE are the intermediate variables, and the beta and T values of the hypothesis H1–H14 test are shown on the arrow line.

#### 5.3.3. Results of the Moderating Effect

This study confirmed the moderating effect of power distance on the relationship between the four aspects of the owner’s safety management and ASB and SSB. Table 5 shows the empirical results of 5000 bootstrap sub-samples. The results show that power distance exhibited a significant moderating effect on the relationship between OC and ASB, SFI and ASB, SFI and SSB, and SS and SSB, which were represented by H16c (β = 0.057, T = 3.237, *p* < 0.01), H16e (β = 0.051, T = 2.806, *p* < 0.01), H16f (β = 0.034, T = 2.786, *p* < 0.01), and H16h (β = 0.19, T = 1.963, *p* < 0.05), respectively. The moderating effect on H16c and H16h was negative, while that on H16e and H16f was positive. However, the hypothesis that power distance moderates the effect of other aspects on ASB or SSB was not supported.

**Figure 4:**
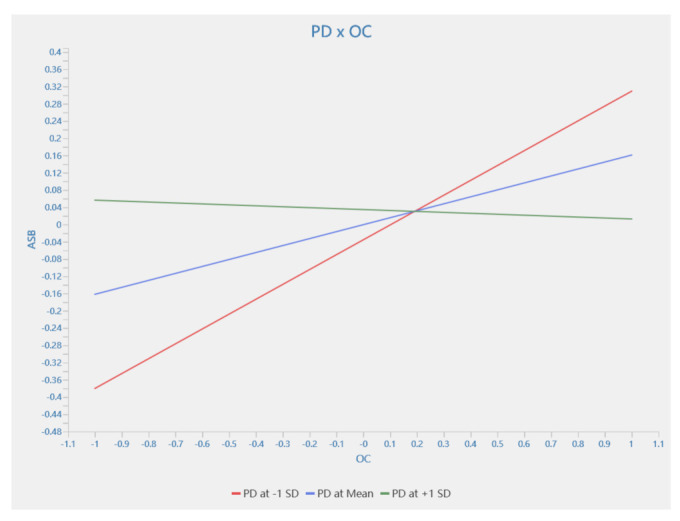
The moderating effect of PD between OC and ASB.

**Figure 5:**
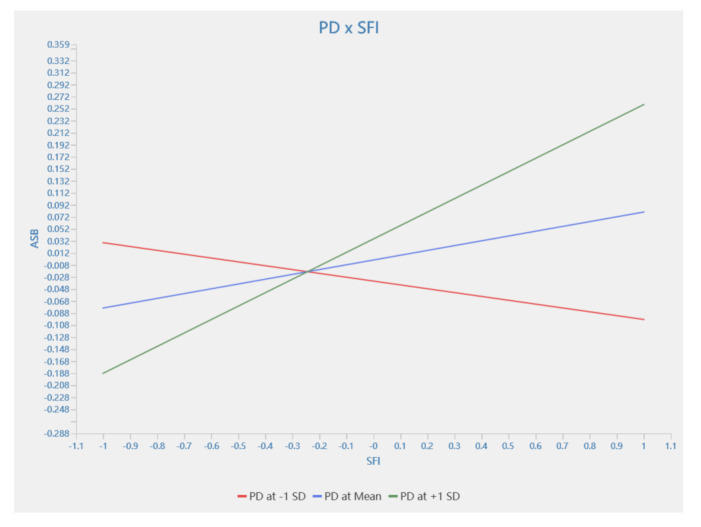
The moderating effect of PD between SFI and ASB.

A simple slope analysis was performed using Smart PLS 4.0 to clarify the moderating role of power distance between the four aspects and ASB and SSB. As shown in Figure 4 and Figure 5, power distance was divided into three levels, namely the average, and the average plus and minus one standard deviation to show the changes in the influence of the owner’s safety management behavior on construction workers’ SCB under different power distance levels. Figure 4 shows that at a low level of power distance, the owner’s organization and coordination stimulated employees’ proactive safety behaviors. In contrast, at a high level of power distance, it did not contribute to the construction workers’ proactive safety behavior. Figure 5 shows that at a high level of power distance, the owner’s sufficient investment in safety funds stimulated employees’ proactive safety behaviors. While, conversely, at a low level of power distance, it did not encourage construction workers’ proactive safety behaviors.

## 6. Discussion and Implications

This section discussed the findings of the survey and the theoretical and practical contributions of this study. It is evident that the safety performance of the construction industry needs to be improved [7,8]. Earlier studies found that owners can significantly influence project safety performance through active participation in project safety management [17]. Similarly, the SCB of construction workers has an important impact on safety within an organization [10,11,12]. Therefore, the owners can further improve the safety performance of the construction industry by encouraging construction workers’ SCB. In accordance with JD-R theory and SOR theory, this study analyzed how owners can provide job resources for construction workers, influence their safety attitude and motivation, and encourage their SCB.

### 6.1. Discussion

Safety objectives had a positive influence on proactive and prosocial safety behavior. The results of this influence are consistent with those of previous studies. Owners’ expectations of safety objectives will form a good organizational safety atmosphere and have a positive impact on workers’ behavior [33,34]. If these objectives are not consistent, it can lead to unclear roles, which has a negative impact on safety behavior [11]. This result confirms the JD-R theory and supports the conclusion of a previous study [55]. Owners should clearly define project safety objectives and tasks to encourage construction workers in high-demanding work environments to work more enthusiastically and commit to their jobs, improve safety performance, use existing job resources to complete the work objectives, and subsequently obtain new job resources.

Furthermore, organization and coordination had a positive effect on proactive and prosocial safety behavior. Good organization and coordinated management can greatly reduce role ambiguity and conflict and improve management at all levels. This, in turn, reduces a possible negative impact on the SCB of construction workers, which is consistent with previous studies [11,64]. At the same time, this study, although partially, supports the study on JD-R theory carried out by the authors of [55]. In other words, it supports the argument that role ambiguity, conflict, and other negative factors reduce employees’ work energy and affect their enthusiasm. In addition, the positive impact of owners’ organization and coordination behavior on construction workers’ active safety behavior is significantly higher than that of prosocial safety behavior. This may be because the owners regularly communicate with the participants about safety issues, make the participants aware of their safety responsibilities and stimulate their safety intentions. This might stimulate workers’ initiative, which is conducive to their proactive safety behaviors such as actively publishing safety proposals and seeking safer working methods.

Safety funding investment had a significant influence on proactive and prosocial safety behavior. This result is similar to those of studies by Hofmann and Morgeso, Bakker et al., and Tsui et al. [54,67,68], which investigated the positive impact of job resources on workers. It was found that the impact of safety funding investment had a significantly higher effect on prosocial than proactive safety behavior. According to the findings of one study [46], prosocial safety behavior is related to emotional commitment, while proactive safety behavior is solely related to employees’ motivation to take initiative regarding safety. Therefore, the result may be attributed to the organization’s regular inspection of safety equipment and safety education and training. These practices increase the safety awareness of construction workers, improve their safety ability, and subsequently influence their prosocial safety behavior, such as helping co-workers, understanding safety policies, and updating procedures. Work autonomy is one of the most important prerequisites for work initiative [67]. Among the four questions on safety funding investment, the construction workers’ score on the statement “Reward construction workers who find hidden dangers in reported accidents” was significantly lower than that of the other three statements. This may be the reason for the low impact of current safety funding investment on proactive safety behavior. By developing an efficient reward mechanism, owners can support the initiative of construction workers, encouraging them to behave in a safe manner, put forward safety-related suggestions, and seek safer working methods.

Moreover, safety supervision also had a positive effect on proactive and prosocial safety behavior. This result supports SOR theory, which states that environmental stimuli affect the safety attitude and motivation of people in an organization. Safety monitoring can increase workers’ SCB because it influences construction workers’ perceptions of the safety policies, regulations, and procedures of an organization [72]. This result also supports other similar studies. For example, the authors of [68] believed that an environment oriented towards safety positively affects an individual’s safety motivation. In addition, the authors of [40] believed that organizational support of participation in safety procedures and measures and a positive atmosphere towards safety have a positive impact on SCB.

Work engagement played an intermediary role in the influence of owners’ safety management behavior on construction workers’ SCB. Work engagement also played a mediating role in many studies on proactive behavior, e.g., [20,28]. The results of this study confirmed the mediating role of work engagement in Wilmar’s extended JD-R model [62]. When owners provide sufficient safety resources, it can increase work engagement, which is conducive to encouraging construction workers to take initiative and exhibit SCB.

Finally, power distance played a moderating role in the influence of owners’ safety management behavior on construction workers’ SCB. The study found that high power distance did not contribute to the development and encouragement of construction workers’ SCB, which is similar to the results of a previous study [84], in which the authors found that high power distance hinders workplace communication. The results show that, at present, owners of construction companies listen less to their workers, they show little concern for their workers, and workers themselves only listen to the decisions of their superiors. It was found that the scores for SCB statements such as “I will speak freely on safety issues, even if others do not agree” and “I will actively improve and perfect safety policies and procedures” were significantly lower than those for other statements. This indicates that construction workers have inhibitions and respect for authority in a high-power distance environment, making it difficult for them to take the initiative in exhibiting and encouraging safety behaviors. However, power distance had a positive moderating effect on the influence of safety funding investment on construction workers’ SCB, which is contrary to the findings of a previous study [85]. High power distance is not necessarily detrimental to the construction workers’ autonomous and safety-oriented work behavior. This may be because construction workers obey the decisions of their superiors, which prompts them to accept and participate in safety training. This can improve their own safety capabilities and responsibility toward maintaining a safe environment. Owners can also hand out rewards for safe behavior, which motivates workers, or punishments for safety hazards. Overall, all of the practices can significantly improve construction workers’ SCB.

### 6.2. Theoretical Contributions

Firstly, this study contributes to the studies on owners’ safety management behavior and proactive behavior. Despite the owner’s primary responsibility toward safety, there is still little research on the owner’s safety management and its influence on construction workers’ proactive behavior. By exploring the influence of owners’ safety management behavior on SCB, this study proposed four comprehensive and important ideas of owners’ safety management (safety objectives, organization and coordination, safety funding investment, and safety supervision) and thus contributed to the research on owners’ safety management and proactive behavior.

Secondly, this study provides a new perspective on improving the SCB of construction workers through owners’ safety management. Previous studies on the influences among proactive behavior of construction workers, employees, and managers were mostly conducted from the perspectives of individual and environmental factors, such as psychological drivers and the safety climate [11,28]. The existing literature on leadership considers different types of leaders [22,23] but often neglects the perspective of owners. Therefore, this study not only expands the scope of SCB research but also broadens the research on the influences of proactive behavior.

Lastly, this study helps to explore the relationship between owners’ safety management behaviors, SCB, and other proactive behaviors. The results show that owners’ safety management behaviors (including safety objectives, organization and coordination, safety funding investment, and safety supervision) had a positive impact on construction workers’ SCB (including proactive and prosocial behaviors). Work engagement played a mediating role, while power distance played a moderating role. This study confirms the applicability of JD-R theory in investigating safety behaviors [49].

### 6.3. Practical Contributions

#### 6.3.1. Direct Influence of Owner’s Safety Management Behavior on Construction Workers’ SCB

Although the owner may not always have a direct leadership relationship with construction workers when it comes to project safety, the owner’s attitude towards safety and their participation in safety management can have a positive impact on the construction workers’ SCB. Considering the necessity of owners’ safety management and the research results, this study put forward the following suggestions to help owners improve the SCB of construction workers, carry out effective safety management, reduce accidents, and improve safety performance.

Firstly, the owner should formulate the goal of project safety and the policy and strategy of safety management. Owners’ expectations of safety goals can influence workers’ behavior [33,34], clearly define their respective roles and responsibilities, and encourage construction workers to actively and diligently carry out work in a safe manner. The owners should create a community of shared interests under a common goal, set a safety culture tone for the project, awaken workers’ civic consciousness and social responsibility, prioritize the safety of others and the organization, and stop focusing only on their own safety.

Furthermore, owners need to establish a sound safety organization system with clear responsibilities, project safety regulations, and an emergency reward mechanism to awaken the sense of responsibility and obligation of workers towards maintaining safety at the workplace. In order to encourage proactive safety behaviors like safety proposals and spontaneous changes among construction workers as well as prosocial safety civic behaviors like duty awareness and safety communication among workers, owners must also regularly discuss safety issues with participants. The owner should be the organizer of the construction project. Therefore, the owner occupies an important position and exerts a great influence on safe production and management activities. For this to happen, it is necessary to select qualified people who can effectively communicate with and coordinate all participants.

The owner should invest in safety funds and provide sufficient financial support for the construction project and the maintenance and upgrading of safety equipment. To ensure safe behavior, the owner should invest in safety education and training for construction workers. They should also reward workers who discover safety hazards and handle accidents and punish those who do not report them or cause them [107]. By actively carrying out and encouraging construction workers to participate in various forms of safety activities, owners provide opportunities for construction workers to share suggestions about safety and actively participate in safety management. This also encourages workers to pay more attention to the safety of their colleagues and nurtures and strengthens the safety values of construction workers and their attention to SCB. Safety education and training and the use of safety protection facilities improve the construction workers’ safety capabilities, which enables them to assist other workers in completing their work safely.

Finally, owners should strengthen safety supervision to create a positive safety atmosphere. They should improve the inspection management system and regularly hold safety meetings to encourage construction workers to participate in safety procedures, discuss safety accidents and safety hazards, and encourage workers to make suggestions of their own pertaining to safety.

#### 6.3.2. Indirect Influence of Owner’s Safety Management Behavior on Construction Workers’ SCB

Owners’ safety management behavior can affect construction workers’ SCB by influencing their work input. According to JD-R theory, abundant job resources can increase work input. Therefore, owners should provide safety resources and care about the well-being of their workers, recognize and encourage safety participation, adopt sensible suggestions about safety from their workers, and hand out rewards for participation. The support of the organization can encourage the construction workers’ sense of responsibility to the organization. As a result, owners can develop and nurture construction workers’ SCB. Because power distance can influence the relationship between owners’ safety management behavior and construction workers’ SCB, owners and construction workers should maintain the power distance at a relatively low level, but not too low. Power distance usually affects people’s behavior in terms of giving feedback. A high-power-distance environment tends to discourage workers from giving feedback for fear of making a negative impression on their superiors. While, conversely, a low-power-distance environment allows workers to give feedback, which indicates positive behavior and a willingness to progress in their job and communicate with superiors and colleagues [108]. Owners can establish bottom-up confidential feedback channels, listen to workers’ feedback and suggestions on safety, and reduce responsibility for those who take the initiative to report accidents so that workers feel respect from their superiors and develop a closer relationship with people in the organization. Overall, these practices can encourage workers to exhibit proactive safety behavior.

## 7. Conclusions

This study collected data from 534 construction workers and established a structural equation model to explore the influence and mechanism between owners’ safety management behavior and construction workers’ SCB. The research finds that owners’ safety management behaviors (including safety objectives, organization and coordination, safety funding investment, and safety supervision) have a positive impact on construction workers’ SCB (including proactive safety behaviors and prosocial safety behaviors), and work engagement plays an intermediary role in it, and power distance can play a moderating role in it. The theoretical contributions of this study mainly include: (1) This study contributes to the studies on owners’ safety management behavior and proactive behavior. (2) This study provides a new perspective on improving the SCB of construction workers through owners’ safety management. (3) This study helps to explore the relationship between owners’ safety management behaviors, SCB, and other proactive behaviors. The practical contribution is that this study provides a scientific basis and guidance for owners to further improve the safety management level of their construction companies by motivating their workers to exhibit SCB, which is conducive to improving the safety management performance of the whole industry.

However, this study has some limitations and is expected to be improved in the future. Firstly, obtaining responses from all construction workers for this study is unrealistic; therefore, due to time and financial restrictions, a non-probabilistic sampling survey approach is adopted. The data were collected in China. There needs to be a reassessment of the study to see whether the safety management system is applicable in different contexts. More widespread study findings will be attained if an appropriate probability sampling approach can be utilized. If data can be collected from construction companies with different leadership positions, the results of this study will be further tested and expanded. Secondly, it may be considered in the future to set various time points for data collection and to administer questionnaires to the same group of individuals at regular intervals to reduce the deviation in data analysis results. In addition, the results of this study will be further tested and expanded in the future if data can be collected from construction companies under the management of owners with different leadership types, and the exploration of different mediating and moderating variables (such as work stress, organizational culture, etc.) will also enrich the research results in this field. Finally, some of the relationships in this study may have reverse causality. It is suggested that a longitudinal design be conducted in future studies to investigate the reverse causality between variables.

## Figures and Tables

**Table 1:** Measuring items.

Variable	Source	Sub-Dimensions	Items
SMB	[31]	SO	1. The group has overall goals and strategies for project safety work.
			2. The group has detailed and regular safety tasks and objectives.
			3. Management personnel are clear about the safety objectives and work of the entire project.
			4. The group continues to track the progress of security work.
		OC	5. The group regularly communicates safety issues with all participating parties.
			6. All participating management personnel have a willingness to work safely.
			7. All participating management personnel are aware of their safety responsibilities.
			8. All participating management personnel have carried out sufficient safety work.
		SFI	9. Regularly inspect, maintain, and update safety production equipment.
			10. Equipped with safety protection and emergency evacuation facilities and equipment for high-risk construction activities.
			11. Provide sufficient safety education and training to all safety management personnel.
			12. Reward employees who discover and report potential accident hazards.
		SS	13. The group has conducted sufficient safety inspections as planned.
			14. All safety hazards have been rectified or emergency plans have been developed.
			15. The group regularly discusses safety accidents, and those responsible for the accidents have been punished.
			16. Adequate specialized supervision has been provided for high-risk construction activities.
SCB	[16]	ASB	1. I will give advice on work activities related to safety.
			2. I will express opinions on safety matters even if others disagree
			3. I will try to change the way the job is done to make it safer.
			4. I will try to change policies and procedures to make them safer.
		SSB	5. I will help teach new workers the safety procedures.
			6. I will assist others to make sure they perform their work safely.
			7. I will speak up and encourage others to get involved in safety issues.
			8. I will remind my colleagues to follow safety procedures.
			9. I will take action to stop safety violations in order to protect the well-being of other crew members.
			10. I will report crew members who violate safety procedures, and tell new crew members that violations of safety procedures will not be tolerated.
			11. I will actively participate in safety-related meetings, including non-mandatory safety meetings.
			12. I will keep informed of changes in safety policies and procedures.
WE	[78]	-	1. I exert my full effort to do my job.
			2. I am enthusiastic about my job.
			3. I feel positive about my job.
			4. At work, I concentrate on my job.
PD	[93]	-	1. Managers do not need to consult subordinates when making most of the decisions.
			2. Managers should avoid contact with employees outside of work.
			3. Employees should not disagree with the decisions made by management.
			4. Managers should not assign important tasks to employees.

SMB is owner’s safety management behavior; SCB is safety citizenship behavior; WE is work engagement; PD is power distance; SO is safety objectives; OC is organization and coordination; SFI is safety funding investment; SS is safety supervision; ASB is proactive safety behavior; SSB is prosocial safety behavior.

**Table 2:** Demographic profile of the sample.

Demographic Variable	Category	Frequency	Percentage (%)
Gender	Male	415	77.7
	Female	119	22.3
Age (years)	30 and below	119	22.3
	31–40	160	30
	41–50	160	30
	51 and above	95	17.8
Education	Junior high school	26	4.9
	Senior high school	61	11.4
	Junior college	182	34.1
	Undergraduate	205	38.4
	Graduate and above	60	11.2
Tenure (years)	0–1 (including one year)	52	9.7
	1–3	95	17.8
	3–5	105	19.7
	5–10	93	17.4
	10 and above	189	35.4
Position	Civil workers	295	54.4
	Decoration workers	133	24.5
	Urban construction workers	33	6.1
	Others	73	13.5

**Table 3:** Reliability and convergent validity of the measurement model.

Construct	CA	CR	AVE
SO	0.906	0.934	0.781
OC	0.917	0.941	0.8
SFI	0.922	0.945	0.81
SS	0.915	0.94	0.797
ASB	0.896	0.928	0.762
SSB	0.954	0.961	0.757
WE	0.926	0.948	0.819
PD	0.884	0.919	0.74

**Table 4:** Discriminant validity of the measurement model.

Construct	SO	OC	SFI	SS	ASB	SSB	WE	PD
SO	-	-	-	-	-	-	-	-
OC	0.894	-	-	-	-	-	-	-
SFI	0.814	0.738	-	-	-	-	-	-
SS	0.89	0.844	0.852	-	-	-	-	-
ASB	0.735	0.87	0.743	0.887	-	-	-	-
SSB	0.851	0.862	0.89	0.886	0.888	-	-	-
WE	0.676	0.678	0.671	0.675	0.736	0.776	-	-
PD	0.199	0.204	0.236	0.223	0.267	0.175	0.163	-

**Table 5:** Hypothesis testing.

Hypothesis	Path	Original Sample (O)	Sample Mean (M)	Standard Path Coefficient	T-Value	Hypothesis
						Test
H1	SO → ASB	0.254	0.253	0.044 ***	5.712	Supported
H2	SO → SSB	0.208	0.206	0.037 ***	5.625	Supported
H3	OC → ASB	0.214	0.213	0.052 ***	4.131	Supported
H4	OC → SSB	0.144	0.141	0.046 **	3.09	Supported
H5	SFI → ASB	0.286	0.287	0.113 *	2.535	Supported
H6	SFI → SSB	0.219	0.218	0.039 ***	5.66	Supported
H7	SS → ASB	0.171	0.17	0.046 ***	3.721	Supported
H8	SS → SSB	0.173	0.172	0.04 ***	4.37	Supported
H9	SO → WE	0.238	0.24	0.043 ***	5.568	Supported
H10	OC → WE	0.236	0.234	0.053 ***	4.462	Supported
H11	SFI → WE	0.327	0.327	0.044 ***	7.443	Supported
H12	SS → WE	0.152	0.152	0.047 **	3.22	Supported
H13	WE → ASB	0.266	0.271	0.059 ***	4.528	Supported
H14	WE → SSB	0.256	0.262	0.058 ***	4.424	Supported
H15	SMB → WE → SCB	0.221	0.228	0.052 ***	4.247	Supported
H16	PD × SMB → SCB	−0.059	−0.059	0.015 ***	3.891	Supported
H16a	PD × SO → ASB	−0.044	−0.045	0.049	0.908	Not supported
H16b	PD × SO → SSB	−0.042	−0.041	0.038	1.089	Not supported
H16c	PD × OC → ASB	−0.183	−0.183	0.057 **	3.237	Supported
H16d	PD × OC → SSB	0.015	0.016	0.044	0.354	Not supported
H16e	PD × SFI → ASB	0.144	0.143	0.051 **	2.806	Supported
H16f	PD × SFI → SSB	0.416	0.375	0.208 *	2.002	Supported
H16g	PD × SS → ASB	0.071	0.073	0.046	1.542	Not supported
H16h	PD × SS → SSB	−0.094	−0.095	0.034 **	2.786	Supported

* *p* < 0.05, ** *p* < 0.01 and *** *p* < 0.001.

## Data Availability

The data used to support the findings of this study are available from the corresponding author upon request.
